# Case Report: Laser speckle flowgraphy in a patient with uveitis due to immune-related adverse events by immune checkpoint inhibitors

**DOI:** 10.3389/fonc.2025.1492011

**Published:** 2025-05-15

**Authors:** Satoru Kase, Yui Yamashita, Satoshi Takeuchi, Susumu Ishida

**Affiliations:** ^1^ Department of Ophthalmology, Faculty of Medicine and Graduate School of Medicine, Hokkaido University, Sapporo, Japan; ^2^ Department of Medical Oncology, Faculty of Medicine and Graduate School of Medicine, Hokkaido University, Sapporo, Japan

**Keywords:** immune check inhibitor (ICI), immune related adverse effects (irAEs), uveitis, laser speckle flowgraphy, corticosteriod

## Abstract

**Purpose:**

It remains unknown whether choroidal circulation could be altered at the onset of immune checkpoint inhibitor (ICI) uveitis compared with that before ICI treatment. Herein we report a patient with Vogt-Koyanagi-Harada (VKH) disease-like uveitis in the unaffected eye as an immune-related adverse effect (irAE) due to ICIs for metastatic choroidal melanoma who had received enucleation. Moreover, choroidal circulation and choroidal thickness were measured before and after treatment.

**Methods:**

A 58-year-old man had a medical history of enucleation in his left eye due to choroidal melanoma 6 years ago. Metastatic lesions in the gastrointestinal tracts and lung were found, and then he received ICIs three times. About 1 month later, he suffered from blurred vision and metamorphopsia in his right eye. Choroidal circulation was evaluated by mean blur rate (MBR), a relative value showing choroidal blood velocity on laser speckle flowgraphy. Central choroidal thickness (CCT) was measured on optical coherence tomography.

**Results:**

Since ophthalmic findings revealed VKH-like uveitis, oral prednisolone of 30 mg was given for 2 weeks, which were then tapered. MBR was reduced and CCT increased at the onset of ICI uveitis compared with its baseline and resolution after corticosteroid treatment.

**Conclusions:**

Choroidal circulation was disrupted, possibly due to ICI-induced autoinflammatory reaction to the choroid, which was managed by corticosteroid treatment. The combination of MBR and CCT could be a useful biomarker for managing the patients with VKH-like uveitis by ICIs.

## Introduction

Immune checkpoint inhibitors (ICIs) play a key role in treatment options in various unresectable malignancies. Ipilibumab and nivolmab (ipi/nivo) are major ICIs, which involve CTLA-4 and PD-1 among tumor–host immunoreactions (1). ICIs have been considered a major treatment option for patients with advanced melanoma (2, 3). Choroidal melanoma is a common intraocular tumor arising in adults. Enucleation has been one of the major treatment options in Japan, since brachytherapy is uncommon, which is rather common in Eastern and Western countries, though (4). The patients’ prognosis for choroidal melanoma is serious when metastatic diseases occur. ICIs have been recognized as a useful immunotherapy for metastatic choroidal melanoma (5). Since the frequency of choroidal melanoma patients who are treated with ICI after enucleation is likely to increase, ocular oncologists need to share information on managements for such patients.

Although ICI is a new evolutional remedy, there are various side effects related with ICI called immune-related adverse events (irAE). In ophthalmology fields, conjunctivitis and scleritis are the common manifestations, while ICI-induced Vogt-Koyanagi-Harada (VKH) disease-like uveitis (ICI uveitis) is a serious visual disturbance in ophthalmic irAE (6), in which the major symptoms consist of blurred vision and metamorphopsia. However, pathophysiology of ICI uveitis is largely unknown. Laser speckle flowgraphy (LSFG), a blood flow imaging system, is an intraocular circulation measurement tool with non-invasiveness. LSFG utilizes laser scattering to visualize intraocular circulation in two dimensions, which enables ophthalmologists to noninvasively evaluate intraocular circulation in various eyes. The relative value named mean blur rate (MBR) is determined in the macular area, which exclusively reflects choroidal circulation. We have shown that MBR significantly changes in intraocular tumors and its related lesions such as retinal hemangioblastoma (7), optic disc melanocytoma (8), and radiation retinopathy due to choroidal melanoma (9). Moreover, we demonstrated that MBR increased with decreased central choroidal thickness (CCT) following corticosteroid therapy in VKH disease (10); however, there is no report on LSFG application looking at choroidal circulation in managements of ICI uveitis. The authors wondered if choroidal circulation observed by LSFG could be reduced at the onset of ICI uveitis compared with that before ICI treatment.

Muraki et al. reported a patient with choroidal melanoma who, after enucleation, developed liver metastasis treated with local resection of tumors together with nivo alone without irAE (5). However, there are no reports on ICI uveitis arising in a healthy eye in patients with choroidal melanoma following enucleation in the affected eye. Herein we report a patient with irAE in the unaffected eye following ICIs for metastatic choroidal melanoma who had received enucleation in the affected eye. Further, the goal of this study is to assess choroidal circulation changes and validate MBR/CCT as biomarkers.

## Case report

**Figure 1:**
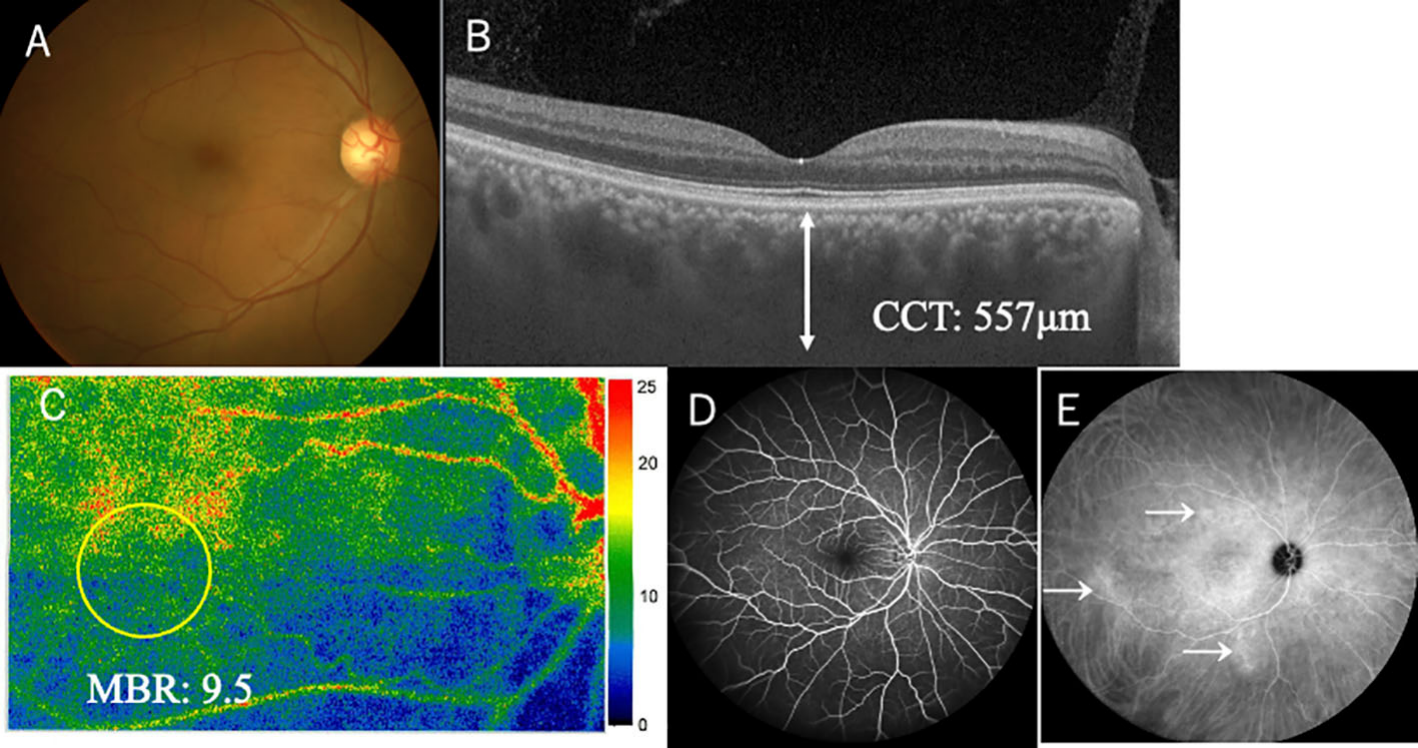
Ophthalmic funduscopy, laser speckle flowgraphy (LSFG), and angiography in right eye 6 years before onset of immune-related adverse effect uveitis. Fundus photography revealed no apparent abnormalities **(A)**. Optical coherence tomography (OCT) depicted relatively thickened choroid in the macula measuring over 500 μm **(B)**. LSFG color map showed normal choroidal circulation in the macula, where mean blur rate (MBR) was 9.5 **(C)**. Fluorescein angiography revealed no abnormalities **(D)**. Indocyanine green angiography **(ICGA)** demonstrated some leakages from the choroidal vessels (E, arrows).

**Figure 2:**
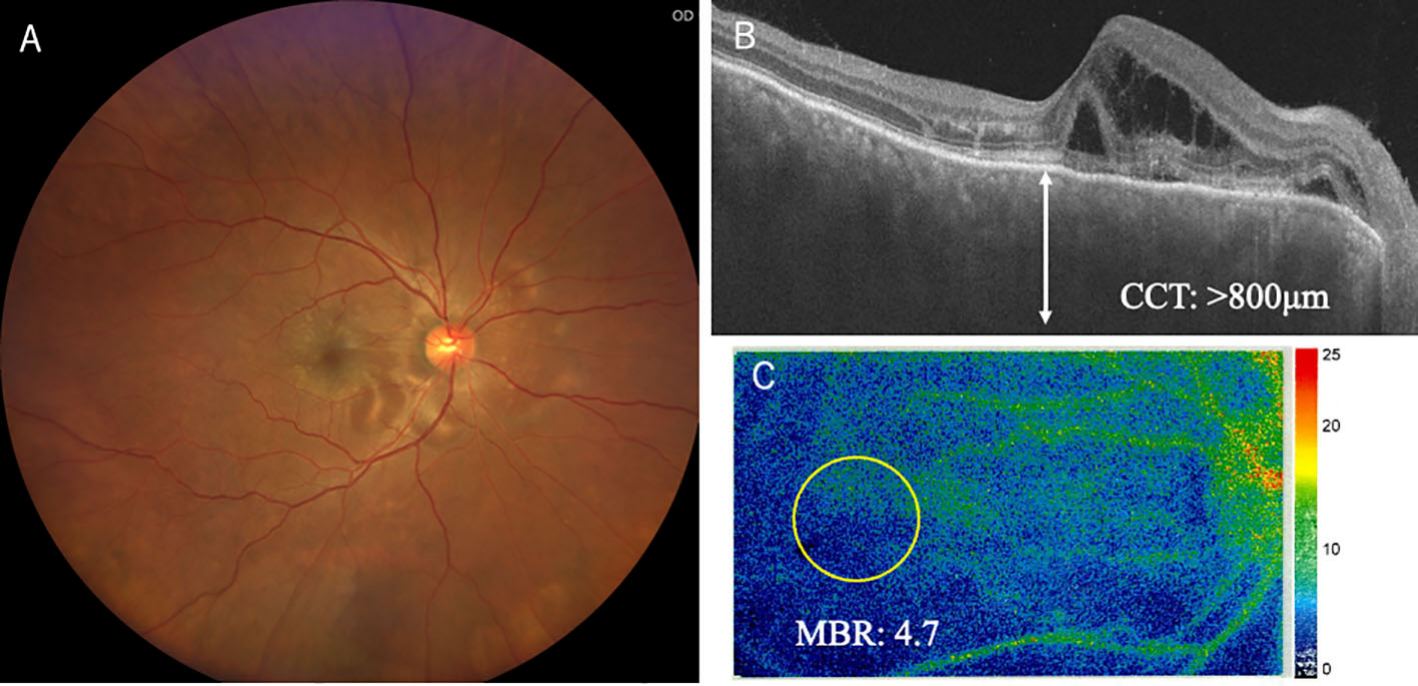
Ophthalmic findings including OCT and LSFG in the right eye at the onset of immune-related adverse effect uveitis. Fundus photography demonstrated lots of serous retinal detachments at the posterior pole and around the optic disc **(A)**. OCT depicted marked thickened choroid, measuring over 800 μm **(B)**. LSFG color map revealed colder coloration, in which MBR was 4.7, compared with 9.5 at baseline **(C)**.

**Figure 3:**
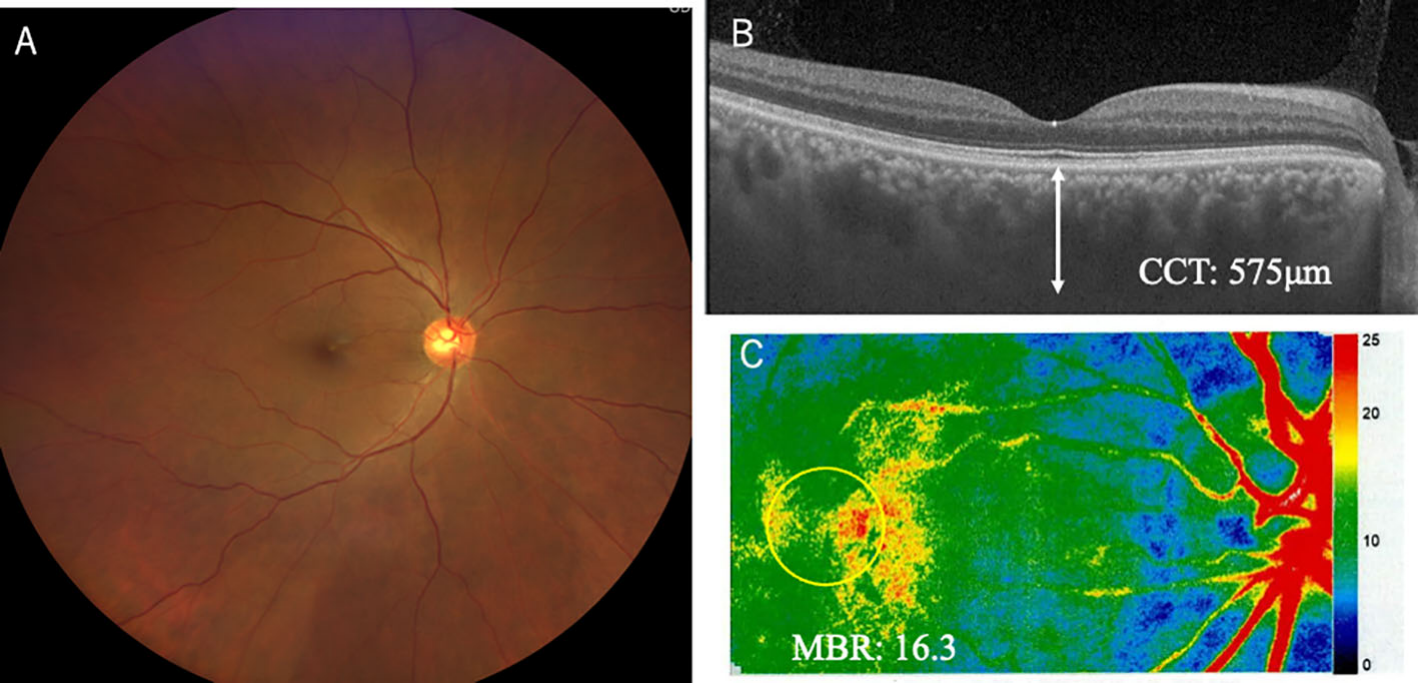
Ophthalmic findings including OCT and LSFG in the right eye 5 months after the onset of immune-related adverse effect uveitis. Fundus photography demonstrated resolution of serous retinal detachment after systemic corticosteroid administration **(A)**. OCT revealed resolution of thickened choroid, measuring around 500 μm, which returned to the baseline level **(B)**. LSFG revealed warmer coloration, in which MBR was 16.3, than that at the onset of uveitis **(C)**.

A 58-year-old man complained of sudden blurred vision in his right eye (oculus dexter, OD). He had a medical history of enucleation of his left eye (oculus sinister, OS) due to choroidal melanoma 6 years ago. We published the clinicopathological data because the tumor could arise from uncomplicated pachychoroid OS (11). With regard to his right eye before enucleation OS, his best-corrected visual acuity (BCVA) was 1.2 OD with normal intraocular pressure (IOP). Slit-lamp examination revealed no abnormality in the anterior chamber and lens. Color fundus photograph (CFP) showed no abnormality in the fundus OD (**Figure 1A**). Optical coherence tomography (OCT) depicted thickened choroid, in which CCT was 557 μm (**Figure 1B**). Fluorescein angiography (FA) revealed no abnormality (**Figure 1D**), while indocyanine green angiography (ICGA) demonstrated mild leakages from the choroidal veins (**Figure 1E**, arrows). These findings indicated that his right eye could be diagnosed with uncomplicated pachychoroid (12). Metastatic lesions had been checked using positron emission tomography-computed tomography (PET-CT) every year. However, PET-CT depicted suspicious metastatic lung tumors in December 2023, and gastrointestinal endoscopy revealed pigmented lesions in the stomach. The biopsy from the latter lesions proved metastatic choroidal melanoma. Based on these findings, ipi/nivo was intravenously given two times, and then he suffered from blurred vision, fever, neck pain, general fatigue, tremor of figures, and erectile dysfunction. Ten days after the third injection, he seriously struggled with impaired vision and metamorphopsia OD and visited our ophthalmology outpatient clinic. BCVA deteriorated to 0.5 with normal IOP. There was neither hyperemia nor anterior chamber inflammation. The lens and vitreous were clear. CFP demonstrated multiple serous retinal detachments (SRD) around the posterior pole OD (**Figure 2A**). OCT depicted not only SRD but also intraretinal fluids and bacillary layer detachments (**Figure 2B**), indicating severe choroidal inflammation. The choroid was markedly thickened, where CCT was over 800 μm (**Figure 2B**). The inflammation was diagnosed with ICI uveitis. His human leukocyte antigen (HLA) typing was HLA-A26 and HLA-DRB1*01 and *09. He initially wished to receive a topical corticosteroid therapy, not systemic therapy, because he was worried about the weakness of ICI effects on the metastatic lesions at this moment. Therefore, sub-Tenon injection of triamcinolone acetonide (STTA) (40 mg) was given; however, his ocular symptoms did not improve. Oral prednisolone (30 mg) was started, which was administered for 2 weeks and then tapered gradually. One month later, his BCVA returned to 1.2 OD, and inflammation was resolved. Six months later, he complained of mild photopsia OD, but CFP and OCT revealed that SRD disappeared (**Figures 3 A, B**). CCT decreased to 575 μm (**Figure 3B**). He now received 5 mg oral prednisolone every day. ICIs were not given again thereafter, and the irAE has not recurred for 6 months.

**Figure 4:**
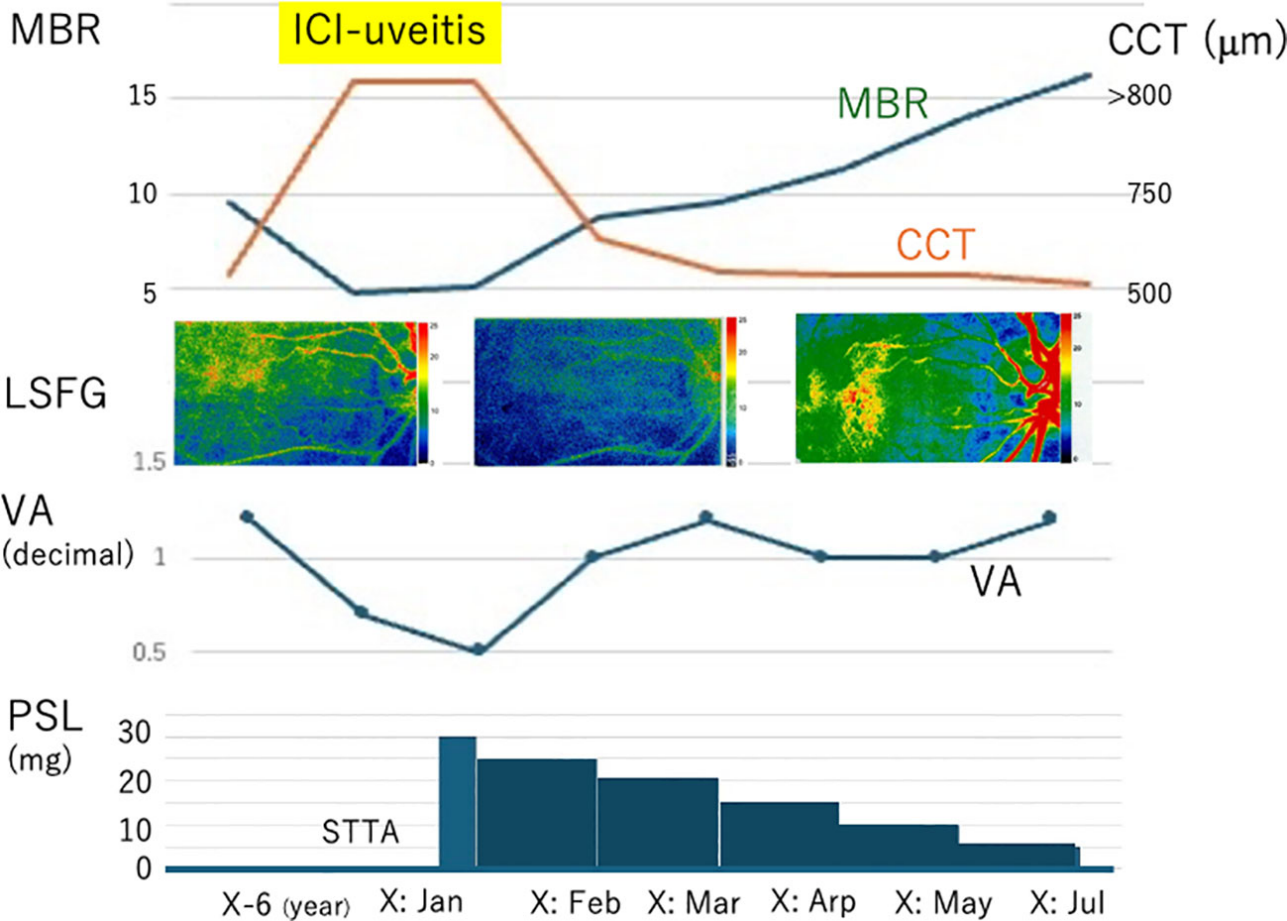
Summary in changes of MBR, CCT, and decimal visual acuity (VA) together with prednisolone (PSL) administrations. MBR decreased, CCT thickened, and VA deteriorated at the onset of ICI uveitis, compared with those at baseline. After stating PSL administration, MBR increased with reduction in CCT while VA recovered.

This study further analyzed choroidal circulatory changes in choroidal blood flows before and after ICI treatments using LSFG. Relative values of intraocular bloodstream velocity were determined as MBR based on the quantitative measurements using LSFG software (LSFG-NAVI^R^, version 3.1.39.2, Softcare Ltd., Fukuoka, Japan) in accordance with previous reports (10). The MBR values OD are shown in **Figure 4** as follows: 9.5, 4.7, 5.0, 8.7, 9.6, 11.3, 14.1, and 16.3 arbitrary units (AU) at the baseline before the onset of ICI uveitis, the onset, 3 days after STTA, and 1–5 months after the systemic corticosteroids, respectively. The MBR of the right eye decreased immediately after STTA and increased gradually after oral prednisolone (**Figures 1C**, **2C**, **3C**). The CCT values OD are shown in **Figure 4** as follows: 577, >800, >800, 768, 590, 573, 575, and 526 μm at the same evaluation points with LSFG. On the other hand, the simultaneous ocular perfusion pressure (OPP) values OD were 54, 55, 46, 49, 52, 52, 44, and 40 mmHg, demonstrating no significant alterations in the right eye.

## Discussion

Alterations of choroidal structure and circulation in ICI uveitis are largely unknown. This study for the first time demonstrated that CCT was even thicker at the onset than before (i.e., uncomplicated pachychoroid as baseline) and after the resolution of uveitis. Moreover, MBR was reduced to about one-half values at the onset. CCT decreased and MBR increased together with BCVA recovery after systemic corticosteroid administration. Although there were no histopathological examinations in eyes with ICI uveitis, it has been reported that autoreactive T-cells were activated by ICIs, which might infiltrate the uveal tissues and retinal pigment epithelium possibly expressing immune checkpoint molecules, leading to ICI uveitis (13). Moreover, pleocytosis is rarely noted in patients with ICI uveitis, suggesting that intraocular tissues could be specifically targeted rather than systemic organs (13), although this patient did not receive lumbar puncture. The current CCT and MBR alterations suggest that choroidal blood flow velocities were reduced due to circulation disruption, which might be caused by the activated T-cell infiltration and complement accumulation within the lumens of choroidal vessels together with the stroma of thickened choroid, and by subsequent choroidal endothelial damage.

We previously demonstrated that CCT was thickened while MBR was reduced at the onset of VKH disease, in which CCT reduction with increase in MBR was noted following systemic corticosteroid administration (10). These results indicated similar pathophysiological alterations in choroidal circulations in ICI uveitis. However, the previous studies on choroidal circulations in VKH disease had limitations on unknown baseline situations (10), because it was impossible to know CCT and MBR before the onset of uveitis. Therefore, there is a possibility that ophthalmologists could underestimate CCT and MBR recovery; that is, choroidal circulations in some patients with VKH disease might not have completely recovered but indeed were evaluated as complete recovery. Such a discrepancy potentially comes from an unknown baseline status of CCT and MBR. In case of ICI uveitis, it is critical for ophthalmologists to know the baseline status of ophthalmic findings before launching ICI treatment, which allows ophthalmologists to evaluate how the choroidal circulation impairments recovered after corticosteroid administration. Taken together, MBR and CCT could be a useful biomarker to manage patients with ICI uveitis.

The treatment strategy for ICI uveitis could consist of topical corticosteroid injection (STTA or intravitreal injection), systemic corticosteroid administration, and/or discontinuation of ICIs (6) or might include anti-IL-6 and tumor necrosis factor-alpha treatment. This patient had already lost his left eye, and his right eye was affected by ICI uveitis, leading to visual impairments. Thus, the patient strongly hoped to receive urgent topical treatment at the onset of uveitis; we decided to do STTA first and then gave oral corticosteroids. Instead, discontinuation of ICIs would take longer to reduce intraocular inflammation than corticosteroid treatment, and the patient initially did not wish so due to concerns about reduction of treatment efficacy on metastatic lesions.

The susceptibility of the onset and development in ICI uveitis is largely unknown; however, at least two possibilities might be considered: choroidal vascular configurations and HLA typing. This patient demonstrated choroidal vascular hyperpermeability on ICGA with relatively thickened CCT, consistent with uncomplicated pachychoroid (12). The enucleated eye in this patient histologically revealed marked dilated venous lumens, where actin filaments were disrupted in the choroidal vascular endothelial cells (11). These abnormalities might have facilitated extravascular ICI leakages in the non-cancerous choroid, provoked local intensive T-cell reaction, and stimulated cross reactivity to self-antigens (14). The abnormality of choroidal morphology would contribute to the susceptibility to ICI uveitis compared with subjects without the abnormality. Regarding HLA typing, this patient had HLA-A26 and HLA-DRB1*01 and *09. Takeuchi et al. demonstrated that HLA-DRB1*04:05 was involved with VKH-like uveitis by ICI treatment (13), while our patient did not have HLA-DRB1*04:05. However, the previous studies demonstrated that HLA-A26 was significantly correlated with panuveitis such as in Behcet’s disease (15) and HTLV-1-associated uveitis (16). Therefore, prophylactic low-dose systemic corticosteroids should be considered to prevent ICI uveitis when additional ICIs were given for metastatic melanoma in this patient. Takeuchi et al. analyzed nine patients with ICI uveitis, and five out of nine patients had HLA-A26 (13). HLA-A26 might be a potential factor involving the onset of ICI uveitis; however, the most significant limitation is that this is a single case report. Future studies enrolling a large number of patients would be necessary to prove the correlation.

In conclusion, choroidal circulation was disrupted, possibly due to ICI induced autoinflammatory reaction to the choroid, which was managed by corticosteroid treatment. The combination of MBR and CCT could be a useful biomarker to manage patients with VKH-like uveitis by ICIs.

## Data Availability

The raw data supporting the conclusions of this article will be made available by the authors, without undue reservation.
